# Structural Analysis and Deletion Mutagenesis Define Regions of QUIVER/SLEEPLESS that Are Responsible for Interactions with Shaker-Type Potassium Channels and Nicotinic Acetylcholine Receptors

**DOI:** 10.1371/journal.pone.0148215

**Published:** 2016-02-01

**Authors:** Meilin Wu, Clifford Z. Liu, William J. Joiner

**Affiliations:** 1 Department of Pharmacology, University of California San Diego, La Jolla, California, United States of America; 2 UCSD undergraduate program, Marshall College, University of California San Diego, La Jolla, California, United States of America; 3 Center for Circadian Biology, University of California San Diego, La Jolla, California, United States of America; University of Waterloo, CANADA

## Abstract

Ly6 proteins are endogenous prototoxins found in most animals. They show striking structural and functional parallels to snake α-neurotoxins, including regulation of ion channels and cholinergic signaling. However, the structural contributions of Ly6 proteins to regulation of effector molecules is poorly understood. This question is particularly relevant to the Ly6 protein QUIVER/SLEEPLESS (QVR/SSS), which has previously been shown to suppress excitability and synaptic transmission by upregulating potassium (K) channels and downregulating nicotinic acetylcholine receptors (nAChRs) in wake-promoting neurons to facilitate sleep in *Drosophila*. Using deletion mutagenesis, co-immunoprecipitations, ion flux assays, surface labeling and confocal microscopy, we demonstrate that only loop 2 is required for many of the previously described properties of SSS in transfected cells, including interactions with K channels and nAChRs. Collectively our data suggest that QVR/SSS, and by extension perhaps other Ly6 proteins, target effector molecules using limited protein motifs. Mapping these motifs may be useful in rational design of drugs that mimic or suppress Ly6-effector interactions to modulate nervous system function.

## Introduction

Ly6 proteins are endogenous prototoxins found in most animals. They contain three short loops extending away from a hydrophobic scaffold that is anchored together by four conserved disulfide bonds [1–3]. They belong to a superfamily of so-called “three-fingered” proteins that includes snake α-neurotoxins, which often interfere with ion channel function and cholinergic signaling pathways (reviewed in [4]). Similarly, Ly6 proteins have also been found to regulate ion channels and nicotinic acetylcholine receptors (nAChRs). One of the best-studied Ly6 proteins is QUIVER/SLEEPLESS (QVR/SSS or SSS), found in *Drosophila*. Originally identified phenotypically in unmapped mutants that “quiver” in response to anesthesia and that exhibit reduced I_A_ currents [5], the gene encoding SSS was subsequently shown to be required for normal levels of sleep and mapped to the *quiver*/*sleepless* (*qvr*/*sss* or *sss*) locus [6–8]. As its name suggests, loss of *sss* causes loss of sleep, much as in animals that have abnormally low levels of one of the protein’s targets, the Shaker voltage-gated potassium (K) channel [9], or high levels of another target, Dα3 nAChRs [7]. SSS has four primary effects on Shaker channels. It upregulates their protein levels [6–8]; it accelerates their activation kinetics [10]; it slows their C-type inactivation [10]; and it speeds their recovery from inactivation [11]. SSS also antagonizes nAChRs by unknown means [7]. The net result is that SSS acts as a suppressor of both excitability and cholinergic synaptic transmission [6–8, 11].

Like other single domain-containing members of the Ly6 family, SSS is highly processed post-translationally. Its N- and C-termini are both cleaved, and the remaining 98 amino acid protein is tethered to cellular membranes. SSS is also modified by N-linked glycosylation. The resulting sugar moiety is a common feature of many extracellular and intrinsic membrane proteins and is therefore unlikely to contribute to selective interactions between SSS and its target molecules. Instead selectivity is likely to be conferred by the protein-forming portion of SSS. How such selectivity is achieved is unknown, however, and is particularly intriguing considering that the small size of mature SSS limits the surface area through which protein-protein interactions can occur.

To determine which protein motifs of SSS are required to form complexes with and regulate K channels and nAChRs, we conducted a series of structure-function studies. We generated deletion mutants of SSS, expressed them heterologously, and assayed for co-immunoprecipitation with Shaker K channels and Dα3 nAChRs. We also tested whether each deletion mutant was able to suppress the activity of α4β2 nAChRs. Our data suggest that only loop 2 (i.e. the middle finger) of SSS is required for interactions with both classes of target molecules and for regulation of nAChRs. We also demonstrate that SSS normally inhibits nAChR activity by reducing levels of receptor at the cell surface, a process for which loop 2 is also required. Our data point for the first time to a structural motif required for a Ly6 protein to modulate effectors of neuronal activity. Such information may be useful in design of Ly6 mimetics or antagonists with pharmacological utility in treating nervous system dysfunction in which cholinergic signaling plays a role.

## Materials and Methods

### Molecular Biology and DNA constructs

SSS loop deletions were generated by QuikChange PCR mutagenesis of untagged SSS/pcDNA and SSS-MYC/pcDNA [7] using the following primers:

ΔL1 F: 5’ CGCGATCGATTTACTGCTATGCGGCCGCTACCTGCAACGGTTGCTGCGT 3’

ΔL1 R: 5’ ACGCAGCAACCGTTGCAGGTAGCGGCCGCATAGCAGTAAATCGATCGCG 3’

ΔL1K1 F: 5’ TGCTATGAGTGCGACGCGGCCGCTCGCTGCAAGGATCCT 3’

ΔL1K1 R: 5’ AGGATCCTTGCAGCGAGCGGCCGCGTCGCACTCATAGCA 3’

ΔL1K2 F: 5’5’ GATGCCCGCTGCAAGTTGATGACCTGCAAC 3’

ΔL1K2 R: 5’ GTTGCAGGTCATCAACTTGCAGCGGGCATC 3’

ΔL2 F: 5’ AACGGTTGCTGCGTGGCGGCCGCTATGTGTACGTCACAG 3’

ΔL2 R: 5’ CTGTGACGTACACATAGCGGCCGCCACGCAGCAACCGTT 3’

ΔL3 (untagged) F: 5’ TGGTCGATCACGTGTGCATGGCGGCCGCTTTTTGTGAGGAAGATATGTGC 3’

ΔL3 (untagged) R: 5’ GCACATATCTTCCTCACAAAAAGCGGCCGCCATGCACACGTGATCGACCA 3’

ΔL3 (MYC tagged) F: 5’ TGGTCGATCACGTGTGCATGGCGGCCGCTTTTTGTGAGGAAGATATCGAGC 3’

ΔL3 (MYC tagged) R: 5’ GCTCGATATCTTCCTCACAAAAAGCGGCCGCCATGCACACGTGATCGACCA 3’

*De novo* structural models were generated by submitting the FASTA sequences through Robetta Full-chain Protein Structure Prediction at http://robetta.bakerlab.org/. Robetta models were visualized using the free software PyMol (www.pymol.org). Sequence analysis for prediction of signal peptide and post-translational modifications were performed using algorithms found at http://www.cbs.dtu.dk/services/SignalP/, http://www.imtech.res.in/raghava/glycoep/submit.html, and http://mendel.imp.ac.at/gpi/gpi_server.html. HA-tagged α4 was generated as previously described [12]. HA-tagged Dα3 and GFP-tagged Shaker were generated as previously described [7].

### Cell Culture, Immunoprecipitations and Biochemistry

HEKtsa cells ([13] via T. Hoshi lab, University of Pennsylvania) and Cos-7 cells (ATCC #CRL-1651 via J. Trejo lab, UCSD) were maintained in growth media containing Dulbecco’s modified Eagles media (DMEM, Mediatech) supplemented with 10% fetal bovine serum (Omega), 1% penicillin/streptomycin (Mediatech) and 1% L-glutamine (Sigma) at 37°C with 5% CO_2_. All experiments were performed using HEKtsa cells, except Shaker and Dα3 co-immunoprecipitations, which were performed using Cos-7 cells. Cells were transfected using X-tremeGene HP (for HEKtsa) or X-tremeGene-9 (for Cos-7) transfection reagent (Roche) as previously described [14]. Cells were lysed in SDS lysis buffer [10 mM Tris, pH 7.5; 100 mM NaCl; 5 mM EDTA; 1% Triton X-100; and 0.05% SDS with cOmplete protease inhibitor (Roche)] and prepared for immunoprecipitation or Western blotting as previously described [14]. α4-HA surface labeling was performed as previously described [12] using rabbit anti-HA (0.6 μl/ml Rockland) antibodies. For co-immunoprecipitation experiments, rabbit anti-GFP antibody (0.2 μl/ml Life Technologies) was used for Shaker and rabbit anti-HA (Rockland) was used for Dα3. Rabbit anti-GFP (1:500, Life Technologies), mouse anti-HA (1:1000, Covance) and mouse anti-MYC (1:250, Santa Cruz Biotechnologies) were used for Western blotting. Peptide-*N*-Glycosidase F (PNGase F) treatment of cell lysates to remove glycosylation modifications were performed according to the manufacturer’s instructions (PNGase, NEB) with the following modification: cOmplete Protease Inhibitor (Roche) was included during the enzymatic treatment (0.3 μl PNGase) which was performed for 2 hours at 37°C. PNGase-treated samples were run on a 12% gel (NuPage, Life Technologies) to maximize band separation at low molecular weight.

### Immunostaining

HEKtsa cells transiently transfected with MYC-tagged SSS-WT or SSS-loop deletion mutants were stained either before or after cell fixation with anti-MYC antibody (1:100, SantaCruz Biotechnologies) in Optimem (Life Technologies) on ice for one hour. Permeabilized cells were incubated in 0.1% TritonX-100 and 0.1% deoxycholate solution in PBS for 1 minute at room temperature, immediately prior to staining. Coverslips were washed once with Optimem and incubated with Alexa Fluor Goat anti-Mouse 488 (1:600, Life Technologies) in 5% goat serum for 1 hour. Coverslips were washed three times in PBS and incubated with DAPI for 1 minute at room temperature. Coverslips were washed once more with PBS and mounted onto glass slides in 1.5 μl VectaShield (Vector Laboratories).

### FRET-Based Measurements of nAChR Activity

HEKtsa cells were transiently transfected with mouse cDNAs of α4β2 nAChRs consisting of α4/pciNeo and β2-dm/pciNeo (gifts from Henry Lester). Cells were co-transfected with TN-XXL/pcDNA3 (gift from Palmer Taylor), with or without untagged SSS-WT and SSS-loop deletion mutants, and prepared for FRET assay as previously described [7]. Briefly, 24 hours after transfection, cells were re-plated into black 96-well clear-bottom plates (Corning) and pre-treated with 1 μM nicotine (R&D Systems). 20 hours later, growth media was replaced with artificial cerebrospinal fluid (ACSF: 121 mM NaCl, 5 mM KCl, 26 mM NaHCO_3_, 1.2 mM NaH_2_PO_4_H_2_O, 10 mM Glucose, 5 mM HEPES, 3.4 mM Ca^2+^, and 0.65 mM Mg^2+^ [pH 7.4]) and assayed by stimulation with increasing concentrations of epibatidine (R&D Systems). Maximum responses were calculated from concentration-response curves averaged over at lest nine experiments, each performed in triplicate, with results normalized to the maximum response of α4β2 nAChRs without SSS. One-way ANOVA repeated-measures analysis with Dunnett’s multiple comparison post test was used for statistical analysis.

## Results

### SSS Loop Deletions Produce Stable Proteins that Traffic to the Cell Surface

**Fig 1:**
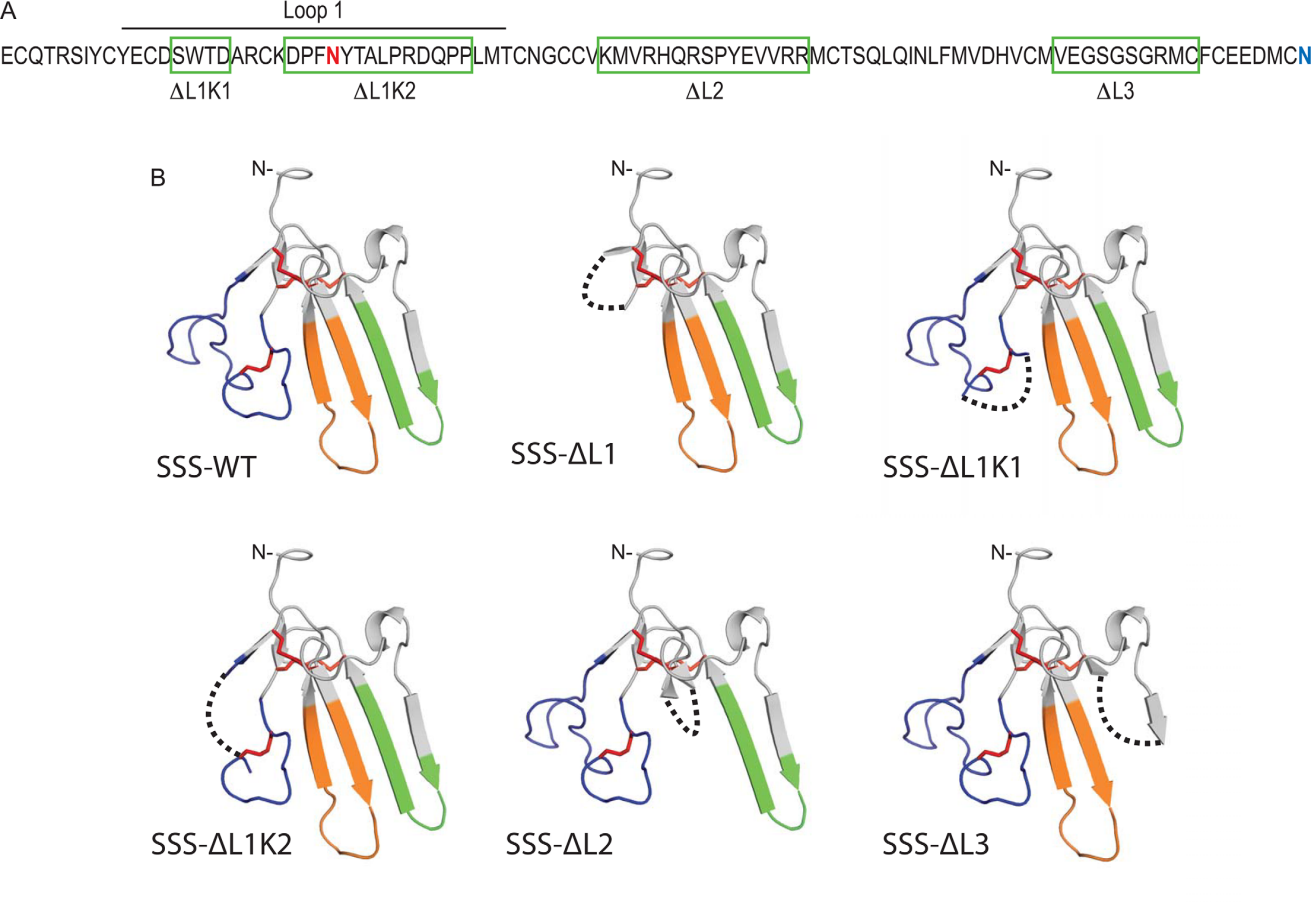
Structural modeling of SSS loop deletion mutants. (A) Amino acid sequence of SSS minus the predicted cleaved N- and C-termini. Loop deletions are designated by green boxes. Predicted N-linked glycosylation site (N57) is highlighted in purple and the predicted GPI-anchor attachment site (N127) is highlighted in light blue. (B) *De novo* Robetta model of SSS-WT. The N-terminus (after cleavage of the signal peptide) is marked accordingly. Red sticks indicate predicted disulfide bonds. Loop regions are denoted in navy (loop 1), orange (loop 2) and green (loop 3). For all panels, deleted protein segments are indicated with a dashed line.

Like Lynx1, the only Ly6 protein whose structure has been solved [3], SSS is predicted to contain a single domain consisting of three short loops extending from a disulfide-rich hydrophobic core. As with other Ly6 proteins, SSS has also been shown to be anchored to the outer leaflet of the plasma membrane by glycophosphatidylinositol (GPI) and to be post-translationally modified by N-linked glycosylation [6, 15–17]. Based on the consensus sequence for the latter type of modification, residue N57 in loop 1 is the most likely site for attachment of the sugar moiety (Fig 1A). Since the only published structural model of SSS is based on an algorithm that used the known structure of an α-neurotoxin as a primer [8], we asked whether *de novo* analysis would support the existing model for SSS. Using the computer program ROBETTA, which employs lowest free energy analysis, we generated an unbiased model of the tertiary structure of SSS that resembles the general structures of other three-fingered proteins (Fig 1B). The only deviation from these other structures is a pair of closely apposed but unbonded cysteines at the position in our model where a conserved disulfide bond is found in other three-finger proteins. Since disulfide bonds normally separate the core from the loops of three-finger proteins, we used the predicted disulfide bonds in our model and the two unpaired cysteines described above to define the beginning of each loop of SSS. We then individually deleted each loop, leaving intact each disulfide-participating cysteine (or similar positions in loop 3) plus one flanking amino acid, and reconnected each exposed end with a three-alanine bridge (Fig 1B). We named these mutants SSS-ΔL1, SSS-ΔL2 and SSS-ΔL3. For each mutant we also made duplicate constructs in which we added a C-terminal MYC epitope. Because subsequent Western blot analysis showed that SSS-ΔL1 expressed poorly (data not shown), we generated two additional deletions that together encompassed all of loop 1, called SSS-ΔL1K1 and SSS-ΔL1K2. The interface between these smaller deletions was defined by an additional predicted disulfide bond, which was preserved in both new mutants (Fig 1B).

**Fig 2:**
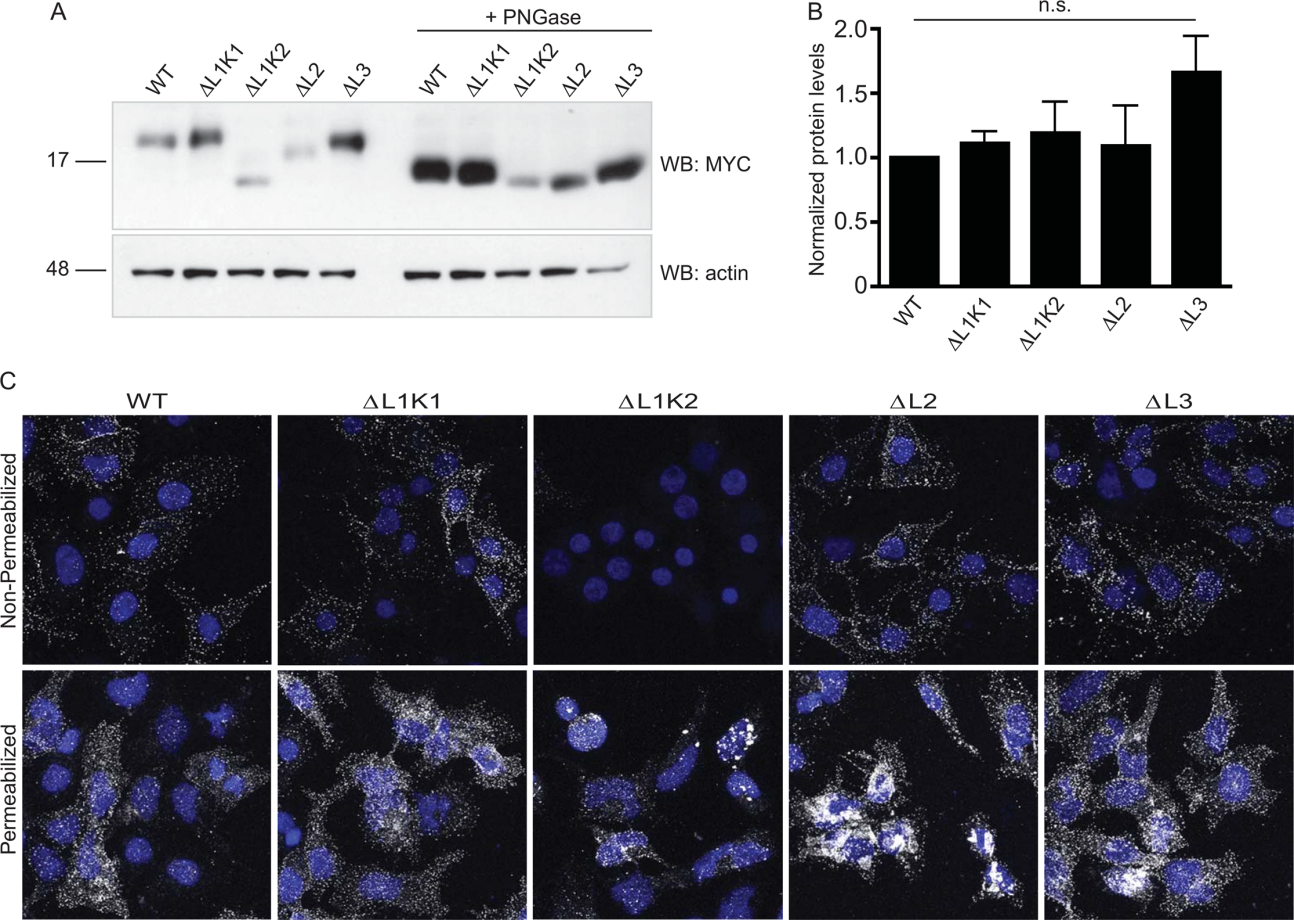
SSS loop deletion mutants are stably expressed and traffic to the plasma membrane. (A) Representative immunoblot of MYC-tagged SSS-WT and loop deletion mutants expressed in transiently transfected HEKtsa cells. Left 5 lanes, untreated total cell lysates; Right 5 lanes, lysates treated with PNGase to remove N-linked glycosylation. Upper panel, immunoblot anti-MYC for SSS detection; bottom panel, immunoblot anti-actin for loading control. (B) Average normalized protein levels of SSS-WT and loop deletion mutants. Protein levels are not significantly different by one way ANOVA using Tukey’s multiple comparison post-test. N = 4. (C) Representative immunostaining of HEKtsa cells transiently transfected with MYC-tagged SSS-WT and loop deletion mutants. Upper panels: non-permeabilized cells show expression of all SSS proteins except ΔL1K1 at the plasma membrane. Bottom panels: permeabilized cells show total SSS expression.

Western blot analysis of cell lysates from transiently transfected HEKtsa cells demonstrated that MYC-tagged SSS-ΔL1K1, SSS-ΔL1K2, SSS-ΔL2 and SSS-ΔL3 produce stable proteins (Fig 2A) at similar levels. Average normalized protein levels over several experiments confirm roughly similar expression levels (Fig 2B). These results support our structural predictions since gross misfolding would be expected to accelerate protein degradation. Furthermore, de-glycosylation with PNGase F (Fig 2A) resulted in the collapse of higher molecular weight bands to lower molecular weight bands, except in the case of SSS-ΔL1K2. We thus conclude that except for SSS-ΔL1K2, which is predicted to lack the sugar moiety of the other constructs, all the loop deletion mutants as well as wild-type SSS (SSS-WT) are modified by N-linked glycosylation.

To test for proper protein folding of each mutant in more detail, we asked whether intracellular trafficking to normal membrane destinations was preserved. To address this question we immunostained HEKtsa cells transiently transfected with MYC-tagged SSS-WT or MYC-tagged loop deletion mutants. Transfected cells were stained with antibody in both non-permeabilizing and permeabilizing conditions, then fixed and imaged by confocal microscopy. Immunostaining of non-permeabilized cells demonstrated that, as previously described [6], wildtype SSS is accessible to antibody on the outside of the cell, as would be predicted for a GPI-anchored protein (Fig 2C; S2 Fig). Furthermore, all of the loop deletion mutants except SSS-ΔL1K2 showed similar expression under the same conditions. Despite the low levels of SSS-ΔL1K2 seen at the cell surface, when transfected cells were permeabilized prior to antibody staining, SSS-ΔL1K2 was present at detectable, although slightly lower levels within the cell. Thus, except for this construct, all mutants undergo normal intracellular trafficking, suggesting that most deletions do not grossly disrupt the structure of SSS.

### Loop 2 of SSS Is Required for Interactions with Shaker and Dα3

**Fig 3:**
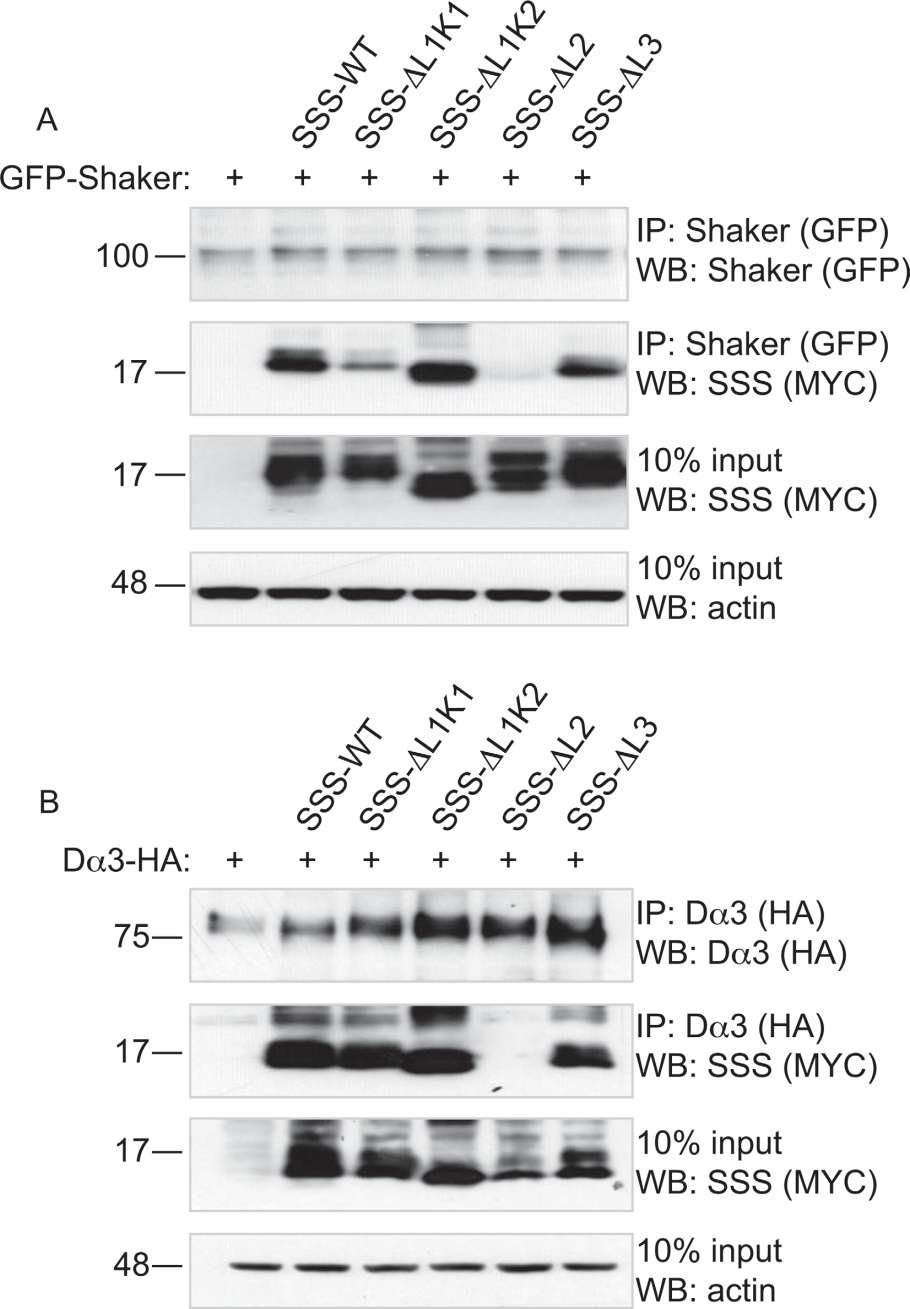
Loop 2 of SSS is required for complex formation with Shaker and Dα3. Representative immunoblots of complexes immunoprecipitated from Cos-7 cells transiently transfected with: (A) GFP-tagged Shaker or B) HA-tagged Dα3, alone or with SSS loop deletion mutants. Top panels: IP-GFP (A) or IP-HA (B), immunoblot anti-GFP (A) or anti-HA (B). Second panels: IP-GFP (A) or IP-HA (B), immunoblot anti-MYC. Third panels: 10% input, immunoblot anti-Myc. Bottom panels: 10% input, immunoblot anti-actin.

Our previous work demonstrated that SSS has a bi-functional role in regulating sleep in *Drosophila*. SSS forms complexes with the Shaker potassium channel and upregulates its levels, activation kinetics and avoidance of C-type inactivation [6, 8, 11]. SSS also forms stable complexes with the Dα3 nAChR and suppresses nAChR function [7]. Collectively, these regulatory processes are thought to suppress excitability and cholinergic synaptic transmission in wake-promoting neurons, thus facilitating sleep in flies. Using our loop deletion mutants, we examined which structural domain(s) of SSS might be involved in interactions with Shaker and Dα3. To address this question we first transiently co-transfected Cos-7 cells with SSS-WT and either GFP-tagged Shaker or HA-tagged Dα3 cDNAs. We then immunoprecipitated tagged channel/receptor and Western blotted for the presence of SSS. As previously reported, we found that wildtype SSS forms stable complexes with both Shaker and Dα3 (Fig 3A and 3B; S3A and S3B Fig). Next we tested which loops of SSS are required for these interactions. We found that SSS-ΔL1K1, SSS-ΔL1K2, and SSS-ΔL3 loop deletion mutants also co-immunoprecipitate with both Shaker and Dα3 to a similar extent as wildtype, with the exception of a slightly lower level for SSS-ΔL1K1 with Shaker. However, deletion of loop 2 (SSS-ΔL2) abolished interactions with both target molecules (Fig 3A and 3B; S3A and S3B Fig). These results indicate that only loop 2 is necessary for SSS to maintain a stable complex with either Shaker or Dα3.

### Loop 2 of SSS Is Required for Functional Inhibition of nAChR Activity

**Fig 4:**
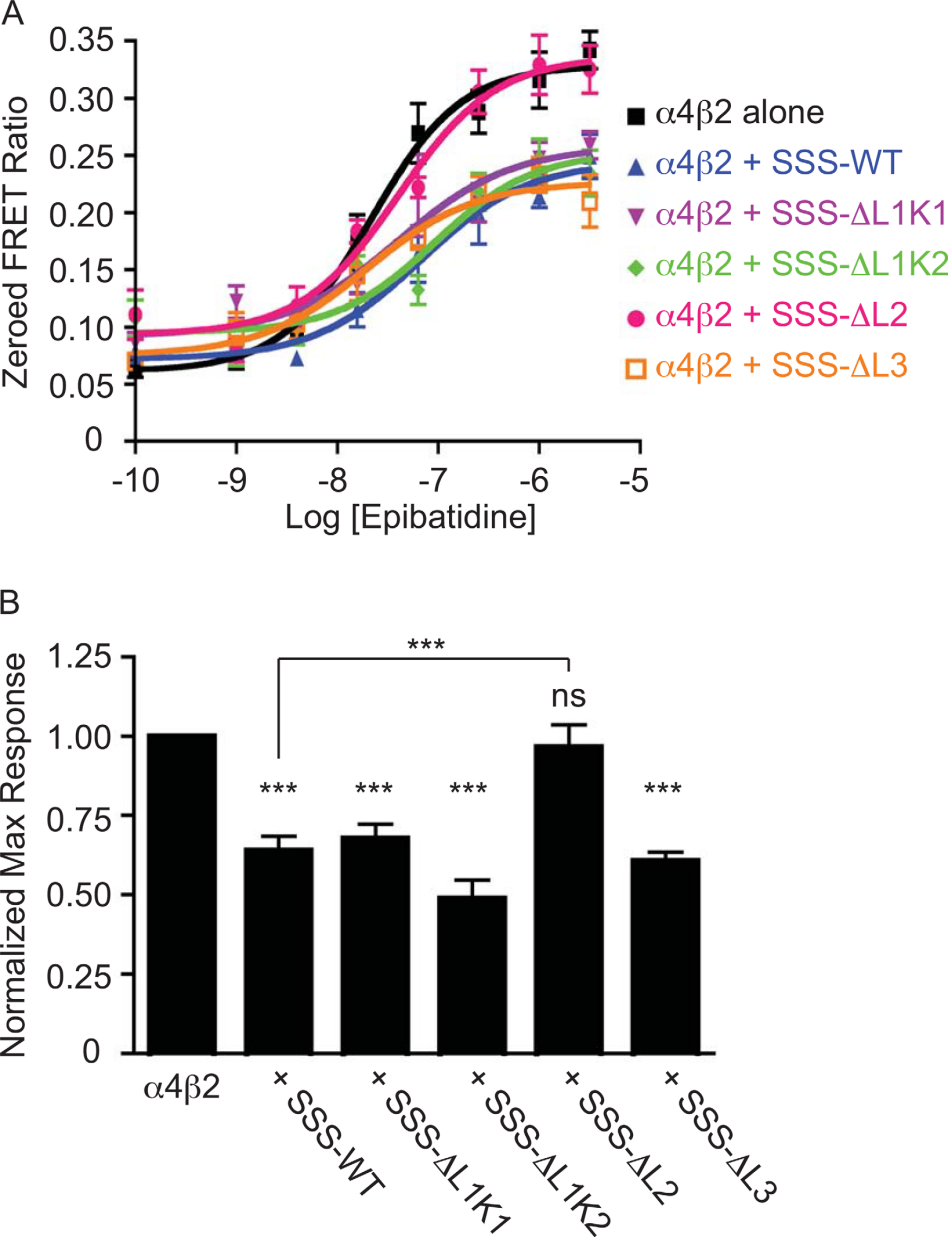
Loop 2 of SSS is required for inhibition of α4β2 nAChR activity. (A) Representative concentration-response curves for α4β2 activation by epibatidine alone (black line) or in the presence of SSS constructs (colored lines). (B) Average normalized maximum responses of α4β2 nAChRs to epibatidine alone or in the presence of SSS constructs (N≥9). ***p<0.001 by one-way ANOVA and Tukey’s multiple comparison post test. All stats are relative to α4β2 alone, except between SSS-WT and SSS-ΔL2, indicated by horizontal bar.

In addition to forming stable complexes with nAChRs, SSS inhibits nAChR activity [7]. To determine if the same part of the SSS protein that facilitates complex formation with nAChRs also facilitates functional regulation of nAChRs, we used the ratiometric FRETable calcium reporter TN-XXL to measure calcium influx following activation of mouse α4β2 nAChRs. We used these receptors for our functional assays because *Drosophila* nAChRs have not produced robust currents alone in heterologous systems. Measurement of FRET ratios in HEKtsa cells co-transfected with α4β2 and TN-XXL after stimulation with increasing concentrations of the nAChR agonist epibatidine resulted in an expected concentration-response relationship for receptor activation (Fig 4A, black curve). As previously reported [7], addition of SSS-WT to transfection mixtures led to a decrease in the maximal response to agonist (Fig 4A, blue curve). Co-expression of all the loop deletion mutants except SSS-ΔL2 also resulted in inhibition of α4β2 activity (Fig 4A, pink curve; Fig 4B). Thus, similar to the structural requirements for interactions of SSS with its known targets, only loop 2 of SSS is necessary for functional inhibition of nAChR activity.

### Loop 2 of SSS Is Required to Reduce Levels of α4β2 nAChRs at the Cell Surface

**Fig 5:**
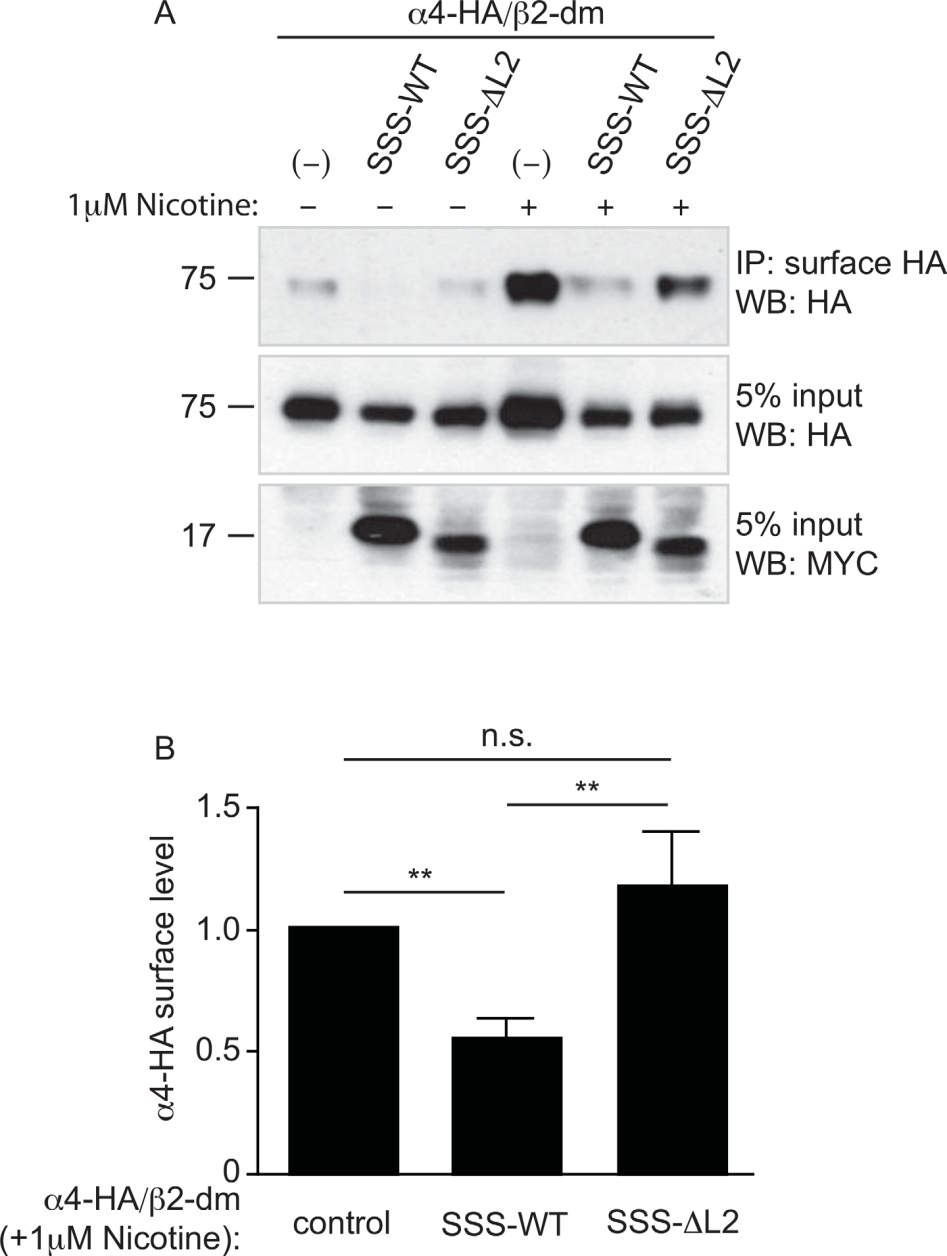
Loop 2 of SSS is required to reduce levels of α4β2 nAChRs at the cell surface. (A) Representative immunoblot of surface-labeled HA-tagged α4 immunoprecipitated from HEKtsa cells co-transfected with untagged β2, alone or with SSS-WT or SSS-ΔL2. Lanes 4–6 are from cells that were pretreated with 1 μM nicotine for 20 hours to enhance trafficking of α4β2 to the cell surface. Upper panel: immunoprecipitated surface α4−HA, immunoblotted with anti-HA. Middle panel: 5% input, immunoblotted with anti-HA. Bottom panel: 5% input, immunoblotted with anti-Myc. (B) Average α4−HA surface levels (normalized to input) in nicotine-treated cells co-transfected with SSS constructs relative to no SSS control. N≥6, **p<0.01 by one-way ANOVA and Dunnett’s multiple comparison post-test.

Like SSS, select mammalian Ly6 proteins have been shown to form complexes with and inhibit nAChR function. Various mechanisms have been proposed to account for the latter effect, including suppressed accumulation of nAChRs at the cell surface [12, 14]. To determine if SSS similarly inhibits α4β2 nAChRs by regulating intracellular trafficking, we measured the amount of α4 subunit at the cell surface in the absence or presence of co-expressed SSS. HEKtsa cells were transiently transfected with HA-tagged α4 and β2, and receptors at the cell surface were labeled with anti-HA antibody prior to cell lysis. Subsequent analysis of the labeled fraction of α4 subunits showed a small but detectable amount of receptor at the cell surface (Fig 5A, lane 1). We also found that surface expression of α4β2 nAChRs was potentiated by pre-incubation of the transfected cells for 20 hrs with the known chaperone, nicotine (1 μM), (Fig 5A, lane 4) as previously described [12, 18–20]. Interestingly, co-expression of SSS-WT substantially reduced the amount of α4 detected at the cell surface, regardless of nicotine pre-incubation (Fig 5A and 5B). These results strongly suggest that like Lynx2 [12], SSS reduces the maximal response of α4β2 nAChRs to agonist stimulation by limiting the availability of nAChR subunits at the cell surface. In contrast, SSS-ΔL2 did not affect surface α4 levels either in the presence or absence of nicotine pre-incubation (Fig 5A and 5B). Since the SSS-ΔL2 mutant reaches the cell surface on its own, our results thus suggest that loop 2 is required to form a complex with α4β2 nAChRs and thus to retain these receptors inside the cell away from activating agonist.

## Discussion

The importance of Ly6 proteins is underscored by their implication in diseases such as Mal de Meleda [21–23]; in nervous system functions such as synaptic transmission, visual plasticity, anxiety, and sleep [7, 14, 24, 25]; and in immune and stem cell functions [16, 23, 26–32]. In some of these cases individual Ly6 proteins have been shown to exert their effects through antagonism of nAChRs, but the mechanisms underlying these effects and the structural motifs that are involved have not been thoroughly investigated. In this study, we examined the contributions of each of three loops of the *Drosophila* Ly6 protein SSS to complex formation with K channel and nAChR effectors, to regulation of nAChR activity, and to trafficking of nAChRs.

Using a *de novo* structural model of SSS based on lowest free-energy folding predictions we identified disulfide bonds that appear to define the first two loops or “fingers” of SSS. This *de novo* model fell just short of predicting the formation of a third disulfide bond at the base of the third loop, despite the presence of conserved cysteines at the appropriate locations. This suggests that although our model is similar to known three-finger structures, some differences do exist, and empirical measurements of the actual structure of SSS will be necessary to validate our predictions.

Our analysis also predicted an additional disulfide bond in the first loop, thus suggesting that SSS structurally resembles non-conventional three-finger toxins, which do not show strong toxicity (reviewed in [4]). We used PCR-based mutagenesis to delete each loop individually as well as to generate two partial, complementary deletions in loop 1. We hypothesized that by leaving the disulfide-rich hydrophobic core of the protein intact we might be able to maintain the stability of each deletion mutant and thereby investigate the contribution of each loop to SSS function. This hypothesis appears to be generally correct. Although the deletion of loop 1 did not form stable protein, we observed stable expression of the remaining mutants, including those with smaller deletions in loop 1 (Fig 2A), suggesting that most mutants were not grossly misfolded or degraded. This hypothesis was reinforced by the PNGase-sensitive migration speeds of each mutant except for SSS-ΔL1K2, which lacked the predicted glycosylation site (Fig 2A), as well as by the detection of all mutants except SSS-ΔL1K2 at the cell surface (Fig 2B). Collectively these data suggest that most deletions do not impair protein stability or trafficking through the secretory pathway from the ER to Golgi to the plasma membrane. These results also validate the general predictions of our structural model for SSS.

Despite the reduction of SSS-ΔL1K2 expression at the cell surface, this deletion mutant as well as the SSS-ΔL1K1, and SSS-ΔL3 mutants are still capable of forming stable complexes with both Shaker and Dα3 (Fig 3) as well as functionally inhibiting α4β2 nAChR activity (Fig 4). Instead, our data indicates that only loop 2 of SSS is necessary for interactions with both channel types and for regulation of α4β2 activity. Interestingly, loop 2 of α-cobratoxin and α-bungarotoxin have been shown to interact with the ligand-binding pockets of acetylcholine binding protein [33] and α1 nAChRs [34], respectively. Thus, our data is consistent with the middle “finger” of three-finger proteins being particularly important for forming complexes with and regulating target molecules. Indeed, molecular modeling and site-directed mutagenesis suggest that the mammalian Ly6 protein Lynx1 interacts with the acetylcholine binding protein via residues at the tip of loop 2 [3, 35]. However, it is somewhat surprising that the same structural element of SSS is responsible for complex formation with both nAChRs and K channels, since these target proteins have very different structures. While nAChRs have large extracellular ligand binding domains with which to interact with Ly6 proteins [36], crystal structures of potassium channels reveal very little protein surface exposed on the outer leaflet of the plasma membrane [37] or its topological equivalent in the vesicular sorting pathway. The shared requirement of loop 2 for interactions with multiple effector molecules has interesting implications for neuromodulatory functions of SSS. For example, it might be expected that a single molecule of SSS would be available to interact with a single K channel or nAChR but not with both simultaneously. This mutually exclusive interaction might hint at a mechanism by which SSS could co-regulate Shaker and Dα3, perhaps acting as a feedback “sensor” of the amount of either channel that is expressed or activated. Alternatively, it is possible that SSS, like some homologous snake α-neurotoxins, might form homodimers (reviewed in [4]) capable of interacting with two effector molecules simultaneously in a tetrameric complex. Further studies will be necessary to determine the likelihoods of both possible scenarios.

Finally, our data reveal a conserved mechanism by which SSS inhibits nAChR function. As we previously showed for the mammalian Ly6 proteins, Ly6h and Lynx2 [12, 14], SSS appears to alter trafficking of nAChRs to reduce receptor levels at the cell surface, thus making them unavailable for activation by agonist (Fig 5). Interestingly, as we also showed for Lynx2 [12], this effect supercedes the ability of nicotine to chaperone α4β2 nAChRs. For SSS, however, we have gone one step further and shown that loop 2 is required to suppress nicotine’s trafficking effects. One possible interpretation of this data is thus that loop 2 outcompetes intracellular nicotine for binding to α4β2 nAChRs. If this were the case then it would also suggest that Ly6 proteins exert some of their regulatory effects by interacting with the agonist-binding site of nAChRs. Such a mechanism would also account for how the SSS-ΔL1K2 mutant, which does not express well at the cell surface, can still interact with and inhibit α4β2 function, since such effects could occur intracellularly.

In conclusion, our data demonstrate both a conserved mechanism for regulation of nAChRs by Ly6 proteins and a structural basis for such modes of regulation. In particular our data suggest a model in which the middle “finger” of some Ly6 proteins interacts with the agonist-binding site of nAChRs to regulate receptor trafficking and ultimately function. In follow-up studies it will be interesting to determine how modes of nAChR regulation attributed to Ly6 proteins such as trafficking, desensitization kinetics, agonist sensitivity and receptor subunit stoichiometry differ in terms of the underlying structural perturbations. Ultimately, potentially subtle differences may be useful in designing drugs with enhanced selectivity for certain nAChR-Ly6 combinations found in restricted regions of the brain and possibly even for specific receptor conformations.

## Supporting Information

S1 Fig Structural coordinates for Robetta model of SSS. (PDB)

S2 Fig Deletion of SSS loop 2 allows for stable protein production but not for trafficking to the cell surface. (PDF)

S3 Fig Full-length Western blots of SSS co-immunoprecipitation data from Fig 3. (PDF)

S1 Table Individual experimental results for Figs 4B and 5B. (XLS)
