# Supplementary material for: Structural Analysis and Deletion Mutagenesis Define Regions of QUIVER/SLEEPLESS that Are Responsible for Interactions with Shaker-Type Potassium Channels and Nicotinic Acetylcholine Receptors
Source: PLoS One. 2016 Feb 1;11(2):e0148215. doi: 10.1371/journal.pone.0148215 (PMC4735452; doi:10.1371/journal.pone.0148215)
Supplement: S2 Fig — (PDF) [file pone.0148215.s002.pdf]

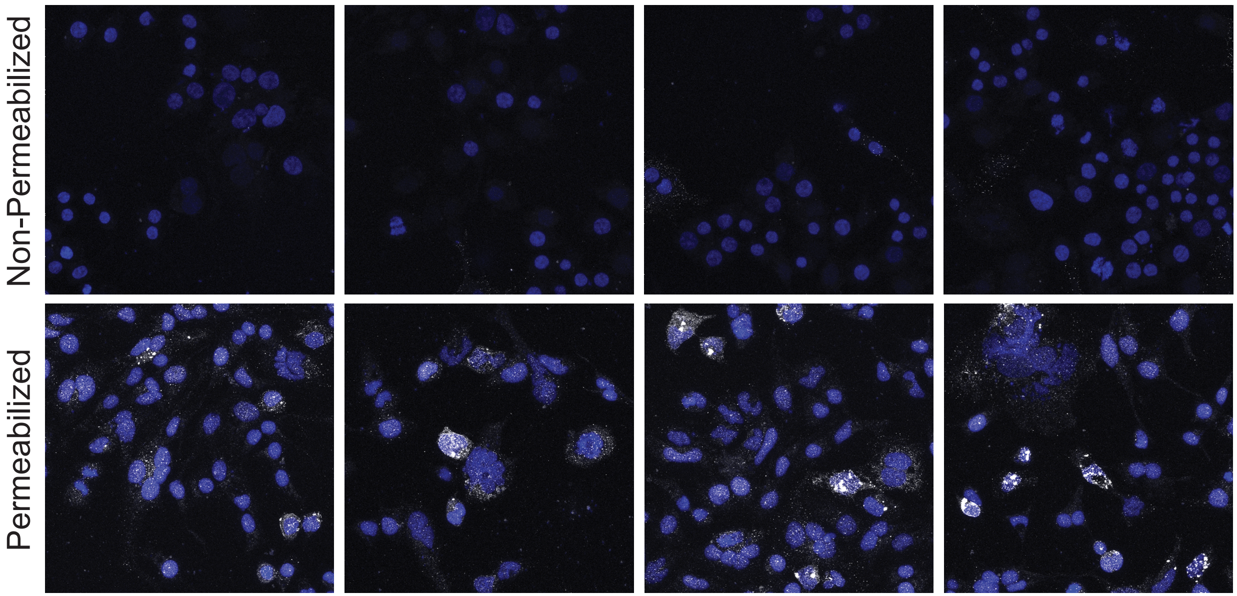

Figure S2. Deletion of SSS loop 2 allows for stable protein production but not for trafficking to the cell surface. This figure presents more examples of cell surface and intracellular staining of SSS ΔL1K2 to complement data in Fig 2c. All panels are from HEKtsa cells transiently transfected with cDNA encoding SSS ΔL1K2. Top four panels are of SSS staining under non-permeabilizing conditions. No protein is apparent at the cell surface. Bottom four panels are of SSS staining under permeabilizing conditions. Protein is present inside cells.
