# Supplementary material for: Structural Analysis and Deletion Mutagenesis Define Regions of QUIVER/SLEEPLESS that Are Responsible for Interactions with Shaker-Type Potassium Channels and Nicotinic Acetylcholine Receptors
Source: PLoS One. 2016 Feb 1;11(2):e0148215. doi: 10.1371/journal.pone.0148215 (PMC4735452; doi:10.1371/journal.pone.0148215)
Supplement: S3 Fig — (PDF) [file pone.0148215.s003.pdf]

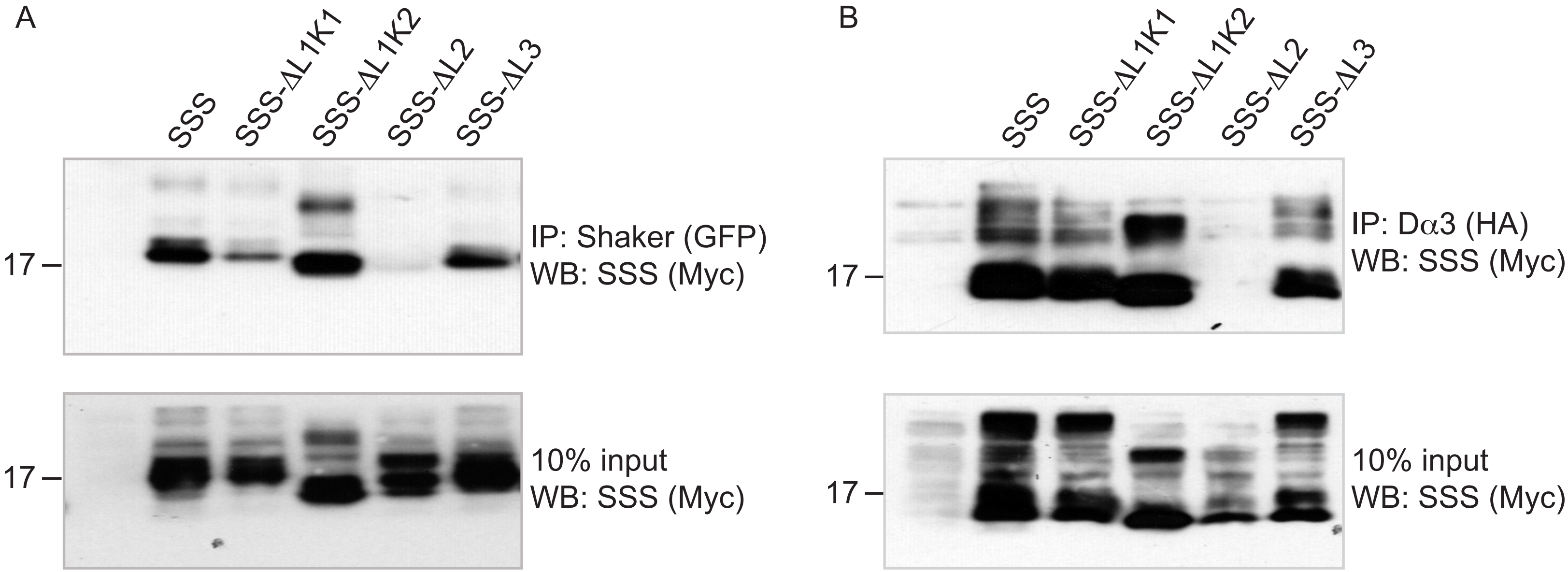

Figure S3. Full-length Western blots of SSS co-immunoprecipitation data from Fig 3.
Top panels: MYC-tagged SSS loop deletions co-immunoprecipitated with GFP-tagged Sh (A) or HA-tagged Dα3 (B).
Bottom panels: 10% input, immunoblot anti-MYC.
